# Supplementary material for: Fast and Accurate Estimation of Selection Coefficients and Allele Histories from Ancient and Modern DNA
Source: Mol Biol Evol. 2024 Jul 30;41(8):msae156. doi: 10.1093/molbev/msae156 (PMC11321360; doi:10.1093/molbev/msae156)
Supplement: msae156_Supplementary_Data [file msae156_supplementary_data.pdf]

Supplementary Information for "Fast and accurate estimation of selection coefficients and allele histories from ancient and modern DNA"

Contents

1 Population genetic models **2**

2 Differing Population Sizes **4**

3 Selection with Dominance **4**

4 Allele Frequency Transition Probabilities Under Dominance **6**

5 Minimum Leaf Flipping Algorithm **7**

6 Simulation Details **7**

6.1 Ancient Genotypes 7

6.2 True Trees 8

6.3 Importance Sampling 8

6.4 Gene Trees on Ancient Data 9

7 Effect of Modern Allele Frequency and Population Size on Inference **10**

8 Supplementary Figures **12**

1 Population genetic models

Consider a Wright-Fisher model with $N(\tau)$ haploid individuals at generation τ and with an infinite sites mutation model with mutation rate μ. Let y_t be the path of a mutation that arose t generations in the past and the allele frequency of the mutation at time τ be $y_t(\tau) \in [0,1]$, $y_t = \{y_t(t), y_t(t-1), ..., y_t(0)\}$. The probability of a path, for a new mutation that starts at frequency $1/N(t)$ at generation t is given by

$$p(y_t) = \prod_{\tau=1}^{t} p_\tau(y_t(\tau), y_t(\tau-1))$$

where $p_\tau(f_1, f_2)$ are the familiar transition probabilities of the Wright-Fisher process of transitioning from allele frequency f_1 in generation τ to frequency f_2 in generation $\tau - 1$. Notice that these transition probabilities are indexed by τ because we consider models with varying population size so that the transition probability matrix may change through time. We consider 1 and 0 to be absorbing states to conform with the infinite sites assumption. Also, these probabilities are implicitly conditional on the age of the mutation, but this is suppressed in the notation. Finally, the probabilities might depend on selection and dominance coefficients that also have been suppressed in the notation. Let Y_t be the set of all possible paths starting at time t, including paths that lead to fixation and loss, such that $\sum_{y_t \in Y_t} p(y_t) = 1$. Then, the probability of a specific path of a mutation, y'_t, given the current allele frequency and given that the age of the mutation is t, is given by

$$p(y'_t|y'_t(0), age = t) = \frac{p(y'_t)}{\sum_{y_t \in Y_t} p(y_t) I(y_t(0) = y'_t(0))}$$

The intensity at which new mutations arise at time t is $\theta(t)/2 = N(t)\mu$, hence, the probability of path y'_t, when not conditioning on the age of the mutation, is

$$p(y'_t|y'_t(0)) = \lim_{T\to\infty} \frac{\theta(t)p(y'_t)}{\sum_{t=0}^{T} \sum_{y_t \in Y_t} \theta(t)p(y_t)I(y_t(0) = y'_t(0)}$$

Similarly, the probability of a path when not conditioning on the current allele frequency, but instead on the event that the mutation has not gone to fixation or loss before the present is

$$p(y'_t|0 < y'_t(0) < 1) = \lim_{T\to\infty} \frac{\theta(t)p(y'_t)}{\sum_{t=0}^{T} \sum_{y_t \in Y_t} \theta(t)p(y_t)I(0 < y_t(0) < 1)}$$

For our purpose, we wish to calculate probabilities of some observed data emitted by the process, either sample allele frequencies or labeled lineages in a coalescence process. Let the observed data at time τ be $x(\tau)$, and the set of all observations for all times be x, $x(\tau) \in x$. We assume that we can calculate

$$p(x|y_t) = \prod_{\tau=0}^{t} p(x(\tau)|y_t(\tau))$$

where $p(x(\tau)|y_t(\tau)) = 1$ if no data are observed at time τ. Then, the desired sampling probabilities are defined as

$$p(x|f) = \lim_{T\to\infty} \frac{\sum_{t=0}^{T}\sum_{y_t\in Y_t}\theta(t)p(y_t)p(x|y_t)I(y_t(0)=f)}{\sum_{t=0}^{T}\sum_{y_t\in Y_t}\theta(t)p(y_t)I(y_t(0)=f)}$$

or

$$p(x|0<f<1) = \lim_{T\to\infty} \frac{\sum_{t=0}^{T}\sum_{y_t\in Y_t}\theta(t)p(y_t)p(x|y_t)I(0<y_t(0)<1)}{\sum_{t=0}^{T}\sum_{y_t\in Y_t}\theta(t)p(y_t)I(0<y_t(0)<1)}$$

where f is the current allele frequency in the population. These two expressions represent different assumptions about the ascertainment of alleles for analysis, where the first expression assumes only alleles of frequency f have been analyzed and the second expression assumes that all variable alleles have been included in the sample. Other ascertainment schemes could also be considered which would then modify the denominator appropriately.

We note that the type of sums given in the numerators and denominators of these expressions can be calculated using the same formalism as used for Hidden Markov Models (HMMs). However, the limits of $T\to\infty$ provide difficulties and the use of a ratio of sums also introduces some inconveniences. An alternative is to work with the reversed process. In fact, for the corresponding diffusion process Nagasawa and Maruyama (Nagasawa and Maruyama, 1979) derived transition densities of the reversed process such that paths of $y_t|y_t(0)$ can be simulated directly backwards in time. A famous result from this model is that $p(y_t|y_t(0), s) = p(y_t|y_t(0), -s)$. This is to say that conditional on the current allele frequency (and assuming a constant selection coefficient and population size), the paths of derived alleles are identical under positive and negative selection and therefore inference methods cannot distinguish between positive and negative selection. The application of Nagasawa and Maruyama's result has been a standard approach for the simulation and inference of allele frequency paths under selection. However, from our standpoint it has two disadvantages. First, we wish to consider models of varying population sizes and varying selection coefficients. For such processes, there are no result similar to those of Nagasawa and Maruyama, which only are valid for a time-homogenous process. Secondly, the derivation of transition probabilities assumes the focal allele is derived, while the true allelic state may be unknown for some real data analysis.

Therefore, we take a different, approximative approach for the transition probabilities of allele frequencies, as described in "CLUES2 Framework" (main text) and "Allele Frequency Transition Probabilities Under Dominance". This approach differs from traditional ways of evaluating the likelihood function using Nagasawa and Maruyama's process in several ways: (1) It allows absorption backwards in time at both allele frequency boundaries of 0 and 1 (rather than only at 0). This allows for the incorporation of

uncertainty in allele polarization and also makes this method robust to allele mispolarization. (2) It uses a finite number of allele frequency bins, rather than allowing allele frequency to vary continuously between 0 and 1. (3) It ignores dynamics before time T. (4) It does not represent a standardized probability measure as it ignores the denominator in the expressions of the sampling probabilities given above, which has the potential to introduce biases as the ignored denominator is a function of the selection coefficient. However, it has the advantage of being easy and fast to evaluate and it can easily incorporate temporal changes in selection coefficients and population sizes. In practice, we will evaluate the likelihood using an HMM starting at $y_t(0) = f$ with unconditional transition probabilities running backwards in time (not to be confused with a formally reversed process) and we will show, using simulations, that this approximation model provides reliable inferences. As we demonstrate through extensive simulations, this approximation provides reliable estimates of selection coefficients and allows for a convenient framework for approximating models with varying selection coefficients and varying population sizes.

2 Differing Population Sizes

We allow the specification of different population sizes in different time periods. This is accomplished by replacing N by a time-specific N_t in both the variance of the normal distribution used for the transition probabilities and the equation for the coalescence emissions. We run a set of ancient genotype simulations, identical to those described in section "Ancient Genotypes" (main text), except that the population size in $[0, 400)$ was $N = 60000$, the population size in the epoch $[400, 600)$ was $N = 200000$ and the population size in the epoch $[600, \inf)$ was $N = 80000$. Figure 1 shows that we can properly account for changes in population size and generate accurate estimates.

3 Selection with Dominance

We allow for arbitrary models of dominance where the relative fitnesses of ancestral allele homozygotes, heterozygotes, and derived allele homozygotes is 1, $1 + hs$, and $1 + s$ respectively. $h = 0.5$ corresponds to additive selection, as described in the main text, while $h = 0$ and $h = 1$ denote a dominant/recessive relationship between the alleles. Of particular note are the cases where $h > 1$ or $h < 0$ as these result in equilibria at derived allele frequency $\frac{h}{2h-1}$. The only change to our approach is to change the mean of the normal distribution used in the transition matrix construction to $x + \frac{s(-1+x)x(-x+h(-1+2x))}{-1+s(2h(-1+x)-x)x}$, which simplifies to $x + \frac{sx(x-1)}{2sx+2}$, as described in the main text, when $h = 0.5$. We demonstrate the ability of this approach to estimate selection coefficients in the presence of different dominance coefficients by running ancient genotype simulations with $s = 0.005$ and dominance coefficients of $h = 0$, 1, and 2. The results of this analysis are shown in Figure S17. We highlight that the variance in the estimator when $h = 0$ is much larger, as low derived allele frequencies are uninformative of s as there will be very few

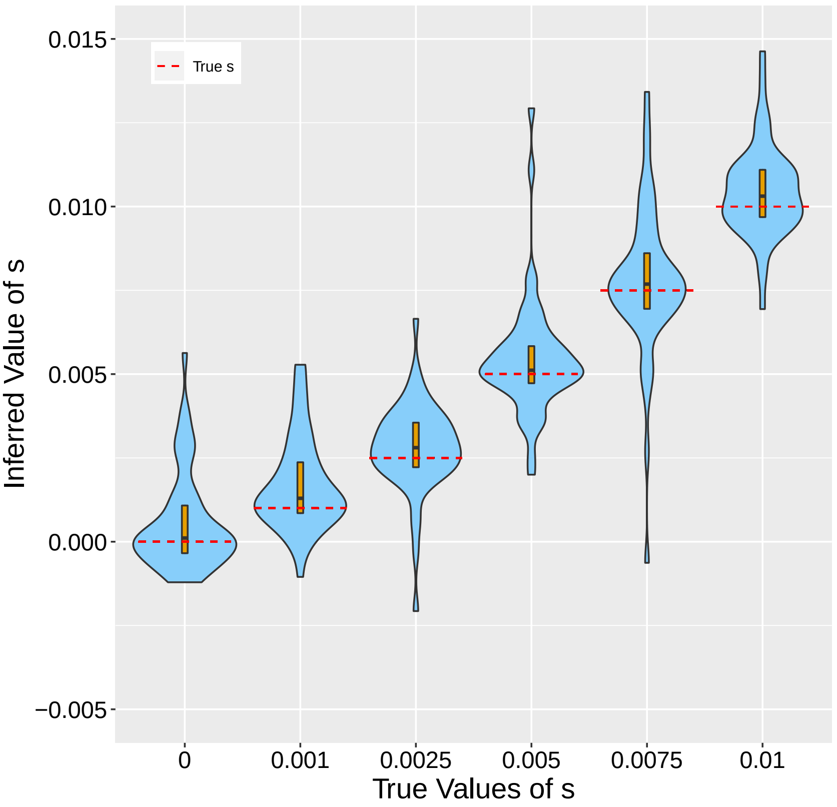

Fig 1: Violin plots showing the results of running CLUES2 on ancient genotype data simulated with changing population sizes through time. Boxplots are overlayed with the whiskers omitted. True values of s are shown as dashed red lines. 50 replicates were performed for each selection coefficient. 2 individuals were sampled in each generation.

homozygous derived individuals in the population. Note that the dominance coefficients are assumed to be constant throughout time and are not themselves inferred. If one wishes to estimate these dominance coefficients, one could do a grid search on the space of coefficients and adjust the degrees of freedom of the corresponding chi-squared distribution appropriately (see the analogous discussion on estimating selection changepoints in Section "Analysis of Ancient Human Data" (main text)).

We apply the method for inference of selection under different dominance regimes to the MCM6 locus. In particular, we run both the pan-ancestry analysis and the ancestry-specific analysis to test whether balancing selection provides a better model fit than the 3-epoch model. For each ancestry, we choose the unique dominance coefficient that generates an equilibrium allele frequency corresponding to the observed modern allele frequency f for that ancestry. This is the value of h for which $\frac{h}{2h-1} = f$. We compare the AIC's generated by each of these analysis to the AIC's of the corresponding 3-epoch model. The results are shown in Table 1. We see that for each ancestry (except ANA, for which there is no evidence of selection at all), fitting a model of dominance provides a much worse fit than the 3-epoch model, which lends support to the hypothesis of changing selective pressures shaping the evolution of the MCM6 locus.

Ancestry	Ancestry-specific modern allele frequency	h	AIC (dominance)	AIC (3-epoch model)
Pan-ancestry	0.535	7.64	-177.32	-297.29
ANA	0.01	-0.01	2.00	3.12
CHG	0.62	2.58	-64.54	-101.68
WHG	0.27	-0.59	-59.49	-69.46
EHG	0.73	1.59	-94.77	-169.88

Table 1: Results of the dominance analysis of the MCM6 locus.

4 Allele Frequency Transition Probabilities Under Dominance

Consider the forward-time Wright-Fisher process of a biallelic locus with alleles A and a where the relative fitnesses of genotypes aa, Aa, and AA are 1, $1+hs$, and $1+s$. If allele A is at frequency x, and assuming Hardy-Weinberg equilibrium, the expected frequency of allele A in the next generation follows the well-known expression

$$\frac{x^2(1+s)+x(1-x)(1+hs)}{x^2(1+s)+2x(1-x)(1+hs)+(1-x)^2} = x - \frac{s(-1+x)x(-x+h(-1+2x))}{-1+s(2h(-1+x)-x)x}$$

By considering the Gaussian approximation to the binomial sampling of the Wright-Fisher model and reflecting the expression for the mean over x, we obtain the expressions seen in Sections "Selection with Dominance" and "CLUES2 Framework" (main text) for the backwards-in-time transition probabilities. We highlight that these expressions are in contrast to the expressions examined in, for example, (Nagasawa and Maruyama, 1979), (Griffiths, 2003), and (Coop and Griffiths, 2004) which consider the time reversed Wright-Fisher diffusion conditional on absorption at allele frequency 0 (which is to say conditional on the allele being derived). The usage of transition probabilities that do not explicitly condition on the allele being derived is by design, as knowledge of the ancestral state may not always be available. When considering gene trees under the infinite site assumption, the polarization is encapsulated in the placement of the mutation on a branch of the tree. In some applications, ARG samplers may allow uncertainty in the ancestral state. In other applications, the ancestral state may have been enforced by the ARG sampler used. However, the impact of using the transition probabilities used in CLUES2 versus conditional transition probabilities is negligible. We show this by performing all simulations with either a forward-time Wright-Fisher simulation or msprime's reverse-time conditional diffusion simulation and observing that using the CLUES2 transition probabilities still results in accurate estimates of the selection coefficients and properly calibrated log-likelihood ratios. In addition, we test the performance of CLUES2 against a modified version of CLUES2 that uses the conditional transition probabilities described in (Nagasawa and Maruyama, 1979), (Griffiths, 2003), and (Coop and Griffiths, 2004), and find that both versions of CLUES2 give nearly identical results (see "Effect of Modern Allele Frequency and Population Size on Inference" for a detailed description of these simulations).

5 Minimum Leaf Flipping Algorithm

We here describe the algorithm we use to process the Relate output to enforce the infinite sites assumption.

Algorithm 1 Minimum leaf flipping algorithm

1. Given tree T with n leaves and allelic state of all leaf nodes
2. Define K as the total numbed of derived leaves
3. For each non-root node v:
   (a) Recursively calculate D_v, the number of derived leaves that are descendants of v
   (b) Recursively calculate A_v, the number of ancestral leaves that are descendants of v
4. For each non-root node v:
   (a) Calculate number of leaf incompatibilities E_v if mutation occurred on the parent branch of v as $A_v + (K - D_v)$
5. Find $\hat{v}$, not necessarily unique, such that $E_{\hat{v}} = \min_w E_w$.
6. Output number of leaf flips $E_{\hat{v}}$ and tree $\hat{T}$, where leaves are now derived if and only if they are descendants of $\hat{v}$

We exhaustively search over all branches in the tree and calculate the number of incompatible leaf states if the derived mutation occurred on that branch. This gives us a list of length $2n - 2$, where there are n leaves in the tree. The minimum number of leaf flips is the minimum value of this list, which is therefore always well-defined. However, there may be multiple nodes that each give the same number of incompatibilities (consider the Newick tree ((A,B),(C,D)); where A and C are derived and B and D are ancestral), so the exact leaves that are flipped are not necessarily unique.

6 Simulation Details

6.1 Ancient Genotypes

We simulate ancient genotype data according to a basic forward-in-time Wright-Fisher simulation with rejection sampling. We begin by sampling a time in the past t_{origin} uniformly on the interval $[0, T_{max}]$ for a large chosen integer T_{max}. Our simulation then begins at derived allele frequency $1/N$, corresponding to a new mutation, at t_{origin} generations before the present. Then, given the frequency at generation k, call it x_k, x_{k+1} is A_{k+1}/N where A_{k+1} is drawn from $Bin\left(N, x_k - \frac{s(-1+x_k)x_k(-p+h(-1+2x_k))}{-1+s(2h(-1+x_k)-x_k)x_k}\right)$. We reject simulations where the derived allele is lost or fixed before we reach the present and retain simulations where the derived allele is still segregating at the present. We now have an allele frequency trajectory that begins at frequency $1/N$ at some point in the past. Given this trajectory, we sample individuals at regularly chosen intervals (e.g. 1 individual every 5 generations, 3 individuals every 1 generation, etc.) and their genotype is recorded. This approach can easily be extended to temporally varying selection

coefficients by simply changing the parameters of the binomial distribution at each timestep. Extending this approach to changing population sizes involves both changing the value of N at each generation as necessary and also drawing t_{origin} in proportion to the population size at each timepoint on the interval $[0, T_{max}]$, rather than uniformly on this interval. This algorithm simulates trajectories unconditional on the age of the allele, but conditional on a current frequency in the open interval (0,1).

6.2 True Trees

For the simulations involving true gene trees under selection, we simulate data using the `SweepGenicSelection` function in *msprime* 1.0, which allows us to quickly and easily simulate trees under genic selection (Baumdicker et al., 2021). As this feature is only available for strictly positive selection coefficients, our simulation results for $s = 0$ were actually simulated with $s = 10^{-6}$, which makes no practical difference to the distribution of trees sampled. All sweep simulations were run with `start_frequency`= $\frac{1}{2N}$ (where here N denotes diploid Ne), which corresponds to a classic hard sweep from a *de novo* mutation. These trees were "recapitated" using the `recapitate` function from the pyslim library (Haller et al., 2019). in order to generate a complete, rooted gene tree.

6.3 Importance Sampling

For our importance sampling simulation study, we use *msprime* to simulate a gene tree under selection as described in the previous section. We then simulate neutral mutations on this tree, using a continuous genome model so that the infinite sites assumption is always satisfied. We also record the true topology of the simulated tree.

We here describe our simple MCMC algorithm. We do not vary the topology of the tree but instead use the true simulated topology. We consider this acceptable for two reasons. The first is that CLUES2 depends only on the coalescence times between allelic classes, resulting in the exchangeability of lineages within allelic classes. Secondly, the lack of recombination and our relatively small sample size (we simulate only 24 leaves) means that there will likely be at least 1 mutation per branch, resulting in only 1 possible topology that is compatible with the infinite sites assumption. Then, given a fixed topology on L leaves, a tree is defined by two sets of parameters:

(1) The vector $(t_L, \ldots, t_2)$ of intercoalescence times, where t_k denotes the time when there are k lineages remaining

(2) The permutation vector $(p_1, ..., p_{L-1})$, an element of the set of permutations on the numbers 1 to $L-1$, which describes the relative ordering of the coalescence events. Note that not all permutations are acceptable, as many of them violate the topological constraints of the tree. For example, a coalescence node cannot occur at an older time than its parent.

We initialize the coalescence times by sampling from the prior distribution on intercoalescence times

(see below). We initialize the ordering of the coalescence events in the tree using the `topo_sort` function from the *igraph* package, which generates a topological sorting of the vertices of a directed acyclic graph (Csardi and Nepusz, 2005). Our target function is:

$$\prod_{k=2}^{L} \frac{\binom{k}{2}}{N} \exp\left(-\frac{\binom{k}{2} t_k}{N}\right) \prod_{b} \frac{(\tau_b \mu L)^{m_b} e^{-\tau_b \mu L}}{m_b!}$$

where we use b to index the branches of the tree, μ is the mutation rate, L is the length of the genomic region, m_b is the number of mutations on branch b, and τ_b is the length of branch b. We use a Metropolis-Hastings algorithm that is inspired by that used by Relate to sample branch lengths (see Supplementary Section 4.2 of (Speidel et al., 2019)) but differs in some minor details. With probability 1/2, we propose a change to the coalescence time vector. We pick a coalescence time t_k uniformly at random and sample a new time from the exponential distribution with mean t_k. With probability 1/2, we choose a pair of adjacent indices in the permutation ordering and propose a switch between them. We automatically reject proposals that violate the topological restraints of the tree. Acceptance probabilities are calculated using the standard Metropolis-Hastings algorithm with our target function. This algorithm, while not efficient, is able to obtain samples from the distribution of branch lengths in a tree with a fixed topology and no recombination events without suffering from the observed problems with existing ARG-inference methods.

6.4 Gene Trees on Ancient Data

Our simulation procedure for generating gene trees on ancient data consists of generating an allele frequency trajectory, sampling the allelic states of modern and ancient samples according to this trajectory, and then simulating the structured coalescent given these samples. We begin by simulating an allele frequency trajectory forward-in-time via the Wright-Fisher model, as described in "Ancient Genotypes". Then, we convert the frequency at each generation into an integer number of individuals by multiplying by N and rounding to the nearest integer. We then initialize our fS derived lineages and our $(1-f)S$ ancestral lineages where S denotes the sample size of modern lineages. Going back in time, we then draw the parent of each derived lineage from the possible derived individuals in the previous generation. We do the same for the ancestral lineages. If 2 lineages have the same parent, then a coalescence is recorded for the appropriate allelic class. We continue this process until our pre-specified time cutoff T. In this way, we obtain a set of derived and ancestral coalescence times, as CLUES2 requires. It is straightforward to incorporate ancient data into this simulation procedure by specifying sampling times $\tau_1, \ldots, \tau_n$, not necessarily unique, and adding an additional derived lineage at time τ_i with probability x_{τ_i} and adding an additional ancestral lineage otherwise. In this way, we can simulate the ancient gene trees input to CLUES2 without having to keep track of the entire tree topology. For the simulations described in the

main text, we have 20 modern haplotypes. For the ancient gene tree simulations, we add 160 leaves to the tree at each of the times 50 and 100 generations before the present. As a comparison, we run identical simulations where we instead sample 80 diploid genotypes at each of the times 50 and 100 generations before the present.

7 Effect of Modern Allele Frequency and Population Size on Inference

We also consider the effect of modern allele frequency and effective population size on inference accuracy. We consider a set of modern allele frequencies $\{0.01, 0.05, 0.3, 0.7, 0.95, 0.99\}$ and a set of effective population sizes $\{100, 1000, 10000, 100000\}$. For each pair of modern allele frequency and population sizes, we simulate 50 true trees from msprime (as described in "True Trees") with the given population size and modern allele frequency. All trees were simulated on 200 leaves and with $s = 0.005$. We plot the inferred selection coefficients for each set of 50 simulations in Figure S18. We find that for low population size ($N_e = 100$), the likelihood function is very flat, causing the optimization algorithm to behave very poorly and resulting in very large estimates of s. However, all of these inferences are accompanied by very low log-likelihood ratios, denoting insufficient evidence for a nonzero selection coefficient. For large population sizes, we see that the inferred selection coefficients concentrate around the true values, although there is higher variance in these estimates for lower modern allele frequencies (particularly frequency 0.01). In Figure S19, we show the log-likelihood ratios against the null $\log \frac{P(D|s^{MLE})}{P(D|s=0)}$, while in Figure S20, we show the log-likelihood ratios against the true parameter value $\log \frac{P(D|s^{MLE})}{P(D|s=0.005)}$. The log-likelihood ratios against the null are small for low modern allele frequencies and/or small population sizes. The log-likelihood ratios against the true parameter value are universally small, indicative that the value of the log-likelihood at the reported MLE is very similar to the log-likelihood at the true parameter value. Occasionally, slightly negative log-likelihood ratios are observed, which are simply due to the optimization algorithm reaching its termination criterion a short distance away from the function optimum. As discussed in "Allele Frequency Transition Probabilities Under Dominance", we also run a set of nearly identical simulations, but with a modified version CLUES2 that uses the allele frequency transition probabilities discussed in (Nagasawa and Maruyama, 1979), (Griffiths, 2003), and (Coop and Griffiths, 2004). These transition probabilities are conditioned on the focal allele being derived. The only difference between CLUES2 and modified version using conditional transition probabilities is that the mean of the Gaussian transition probabilities of the conditional version has a value of $x - 0.5sx(1-x)coth(\frac{1}{2}Ns(1-x))$ (or $x - \frac{x}{N}$ for $s = 0$) instead of $x + \frac{sx(x-1)}{2sx+2}$, as in CLUES2. The results of this analysis are shown in Figures S21, S22, and S23.

References

Franz Baumdicker, Gertjan Bisschop, Daniel Goldstein, Graham Gower, Aaron P Ragsdale, Georgia Tsambos, Sha Zhu, Bjarki Eldon, E Castedo Ellerman, and Jared G et al. Galloway. Efficient ancestry and mutation simulation with msprime 1.0. *Genetics*, 220(3):iyab229, 12 2021. ISSN 1943-2631. doi: 10.1093/genetics/iyab229. URL `https://doi.org/10.1093/genetics/iyab229`.

Graham Coop and Robert C. Griffiths. Ancestral inference on gene trees under selection. *Theoretical Population Biology*, 66(3):219–232, Nov 2004. doi: 10.1016/j.tpb.2004.06.006.

Gabor Csardi and Tamas Nepusz. The igraph software package for complex network research. *InterJournal*, Complex Systems:1695, 11 2005.

R.C. Griffiths. The frequency spectrum of a mutation, and its age, in a general diffusion model. *Theoretical Population Biology*, 64(2):241–251, 2003. ISSN 0040-5809. doi: https://doi.org/10.1016/S0040-5809(03)00075-3. URL `https://www.sciencedirect.com/science/article/pii/S0040580903000753`.

Benjamin C. Haller, Jared Galloway, Jerome Kelleher, Philipp W. Messer, and Peter L. Ralph. Tree-sequence recording in slim opens new horizons for forward-time simulation of whole genomes. *Molecular Ecology Resources*, 19(2):552–566, 2019. doi: https://doi.org/10.1111/1755-0998.12968. URL `https://onlinelibrary.wiley.com/doi/abs/10.1111/1755-0998.12968`.

Masao Nagasawa and Takeo Maruyama. An application of time reversal of markov processes to a problem of population genetics. *Advances in Applied Probability*, 11(03):457–478, Sep 1979. doi: 10.1017/s0001867800032754.

Leo Speidel, Marie Forest, Sinan Shi, and Simon R. Myers. A method for genome-wide genealogy estimation for thousands of samples. *Nature Genetics*, 51(9):1321–1329, 2019. doi: 10.1038/s41588-019-0484-x.

8 Supplementary Figures

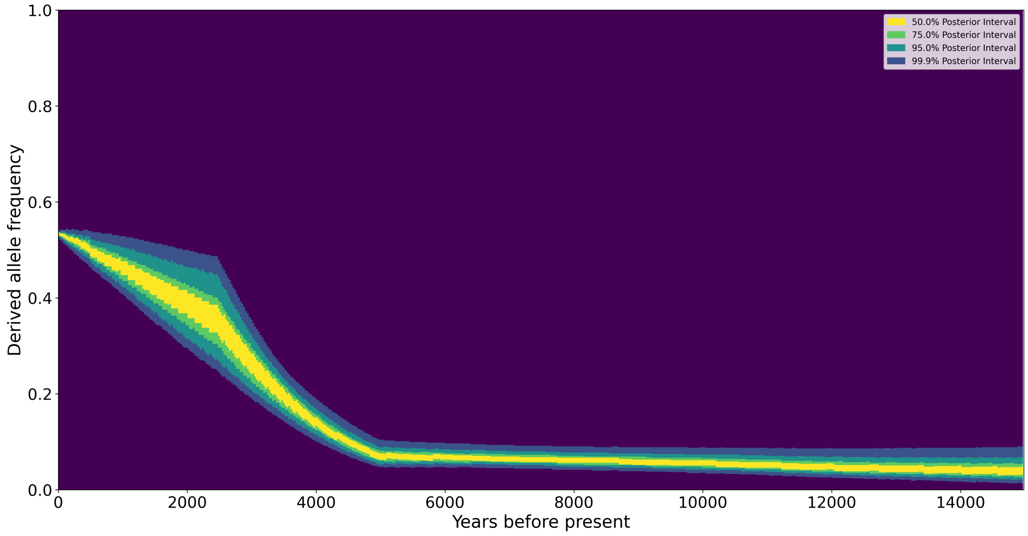

Fig S1: The reconstructed derived allele frequency trajectory for SNP rs4988235 in the MCM6 gene under the 3-coefficient model.

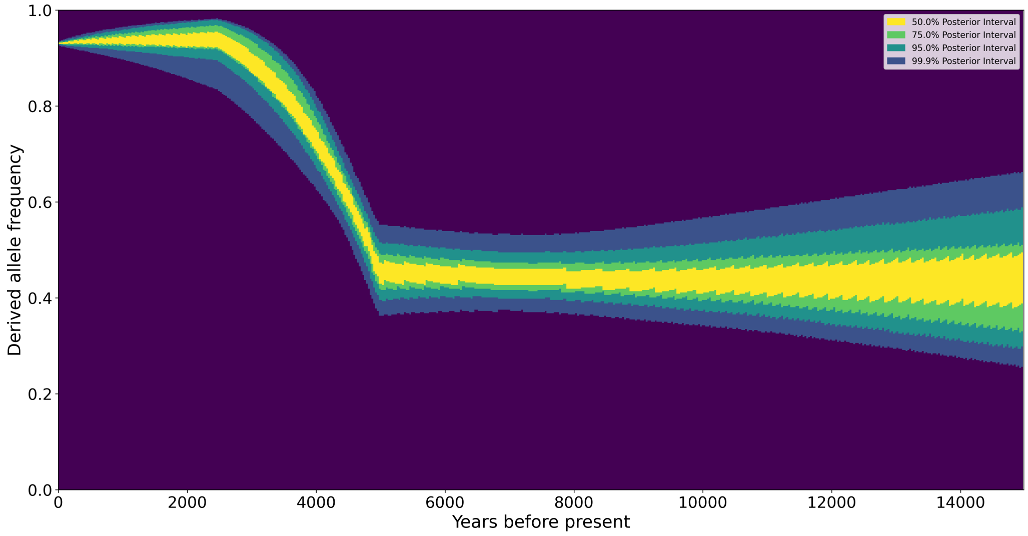

Fig S2: The reconstructed derived allele frequency trajectory for SNP rs35395 in the SLC45A2 gene under the 3-coefficient model.

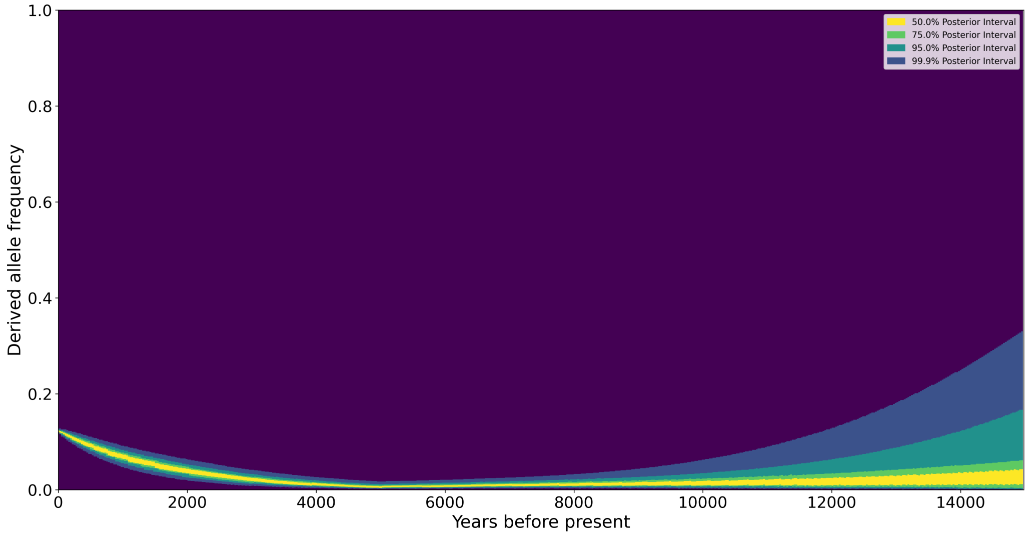

Fig S3: The reconstructed derived allele frequency trajectory for SNP rs12153855 in the TNXB gene under the 2-coefficient model.

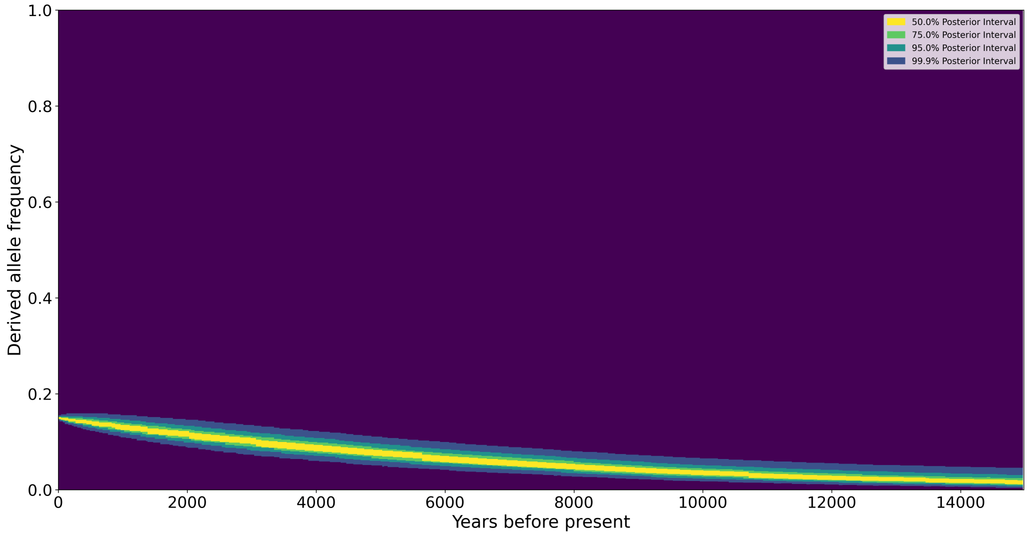

Fig S4: The reconstructed derived allele frequency trajectory for SNP rs75393320 in the ACP2 gene under the 1-coefficient model.

(a) 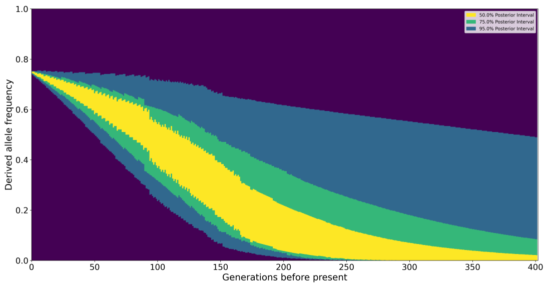 **(b)** 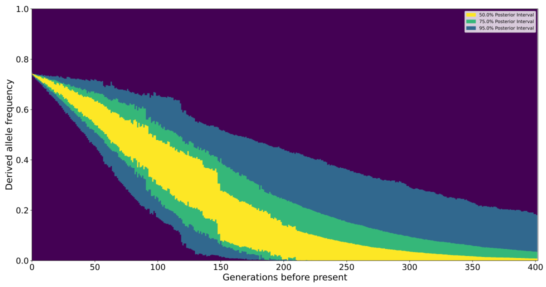

Fig S5: The results of the allele frequency trajectory reconstruction of CLUES2 with (a) the Monte Carlo integration framework and with (b) the exact rejection sampling approach for a 1-selection coefficient model.

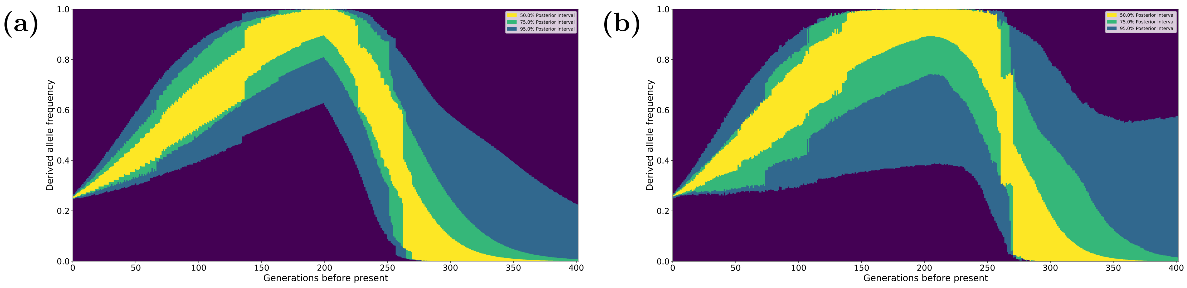

Fig S6: The results of the allele frequency trajectory reconstruction of CLUES2 with (a) the Monte Carlo integration framework and with (b) the exact rejection sampling approach for a 2-selection coefficient model.

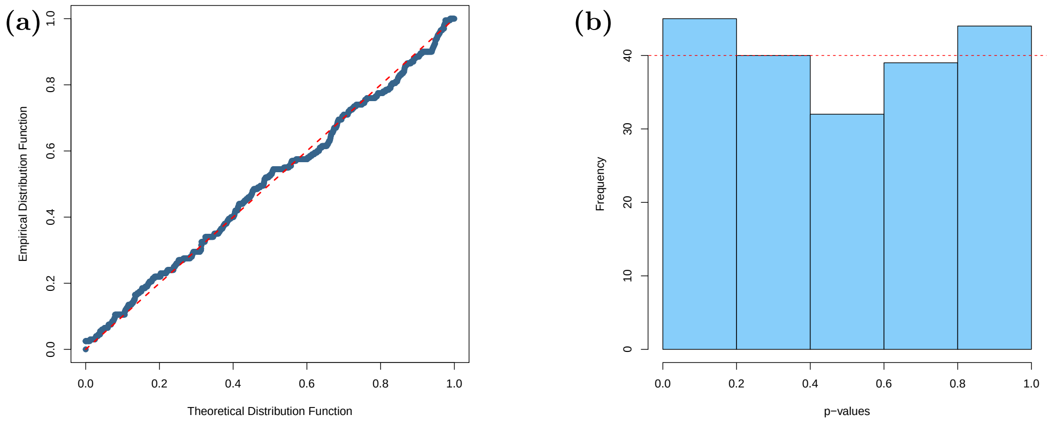

Fig S7: The (a) P-P plot and (b) p-value histogram for our ancient genotype simulations.

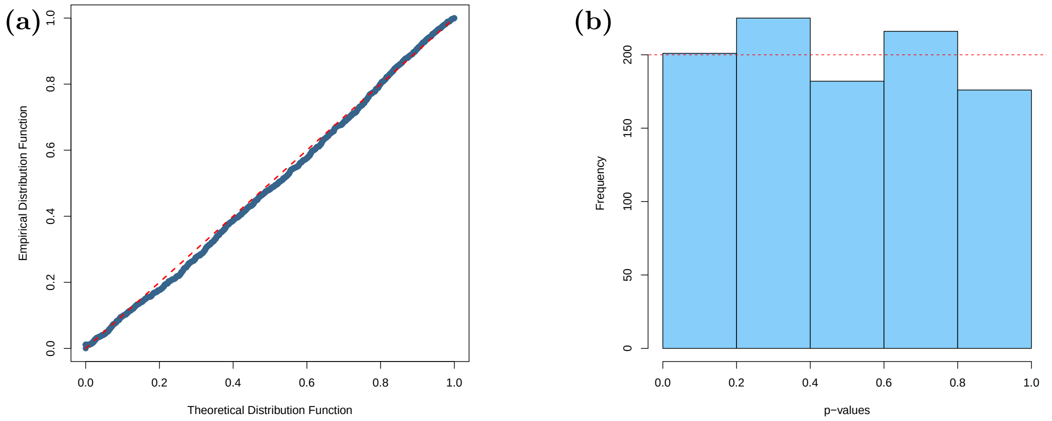

Fig S8: The (a) P-P plot and (b) p-value histogram for our true tree simulations.

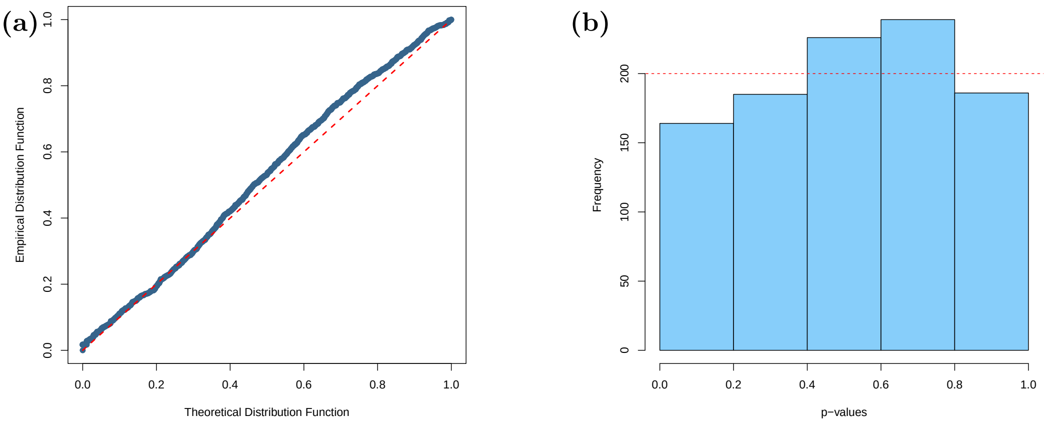

Fig S9: The (a) P-P plot and (b) p-value histogram for our importance sampling simulations.

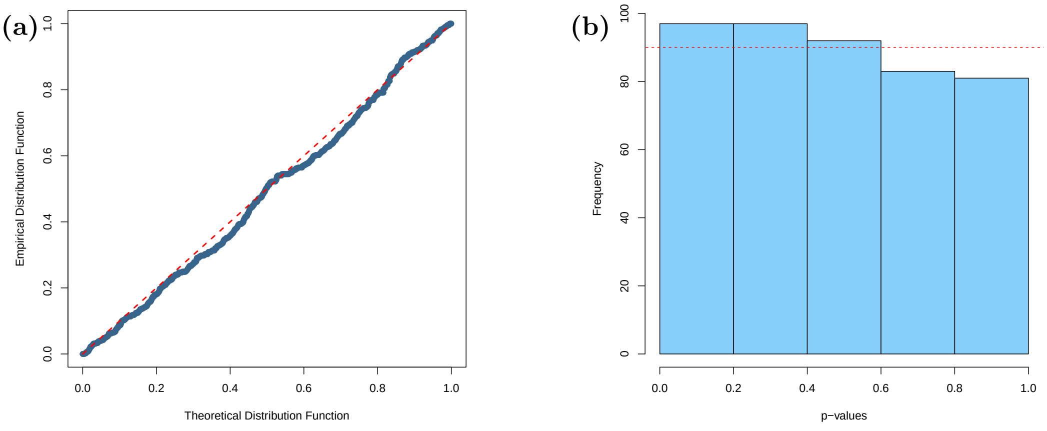

Fig S10: The (a) P-P plot and (b) p-value histogram for our simulations of multiple selection coefficients.

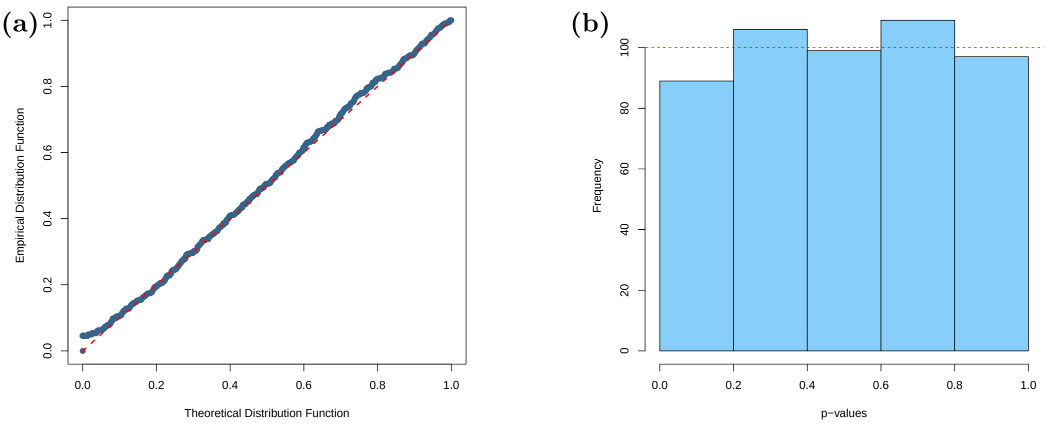

Fig S11: The (a) P-P plot and (b) p-value histogram for our simulations with varying population sizes.

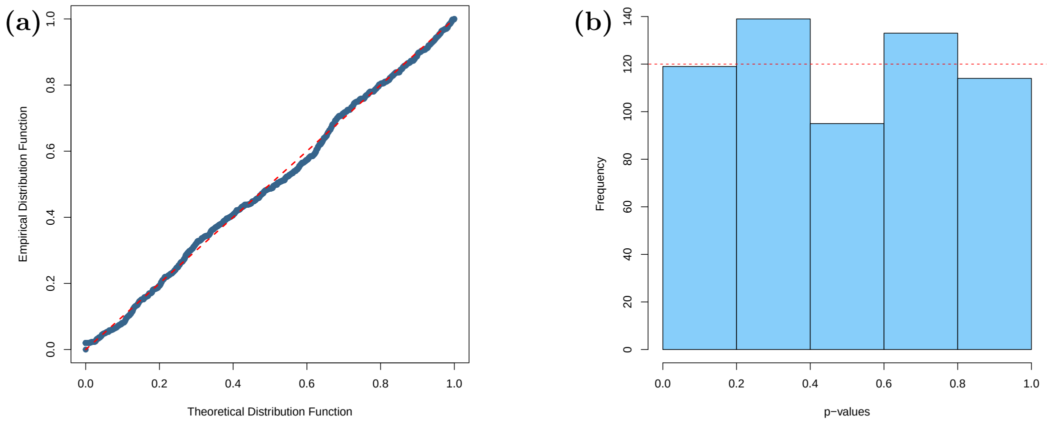

Fig S12: The (a) P-P plot and (b) p-value histogram for our simulations with ancient gene trees.

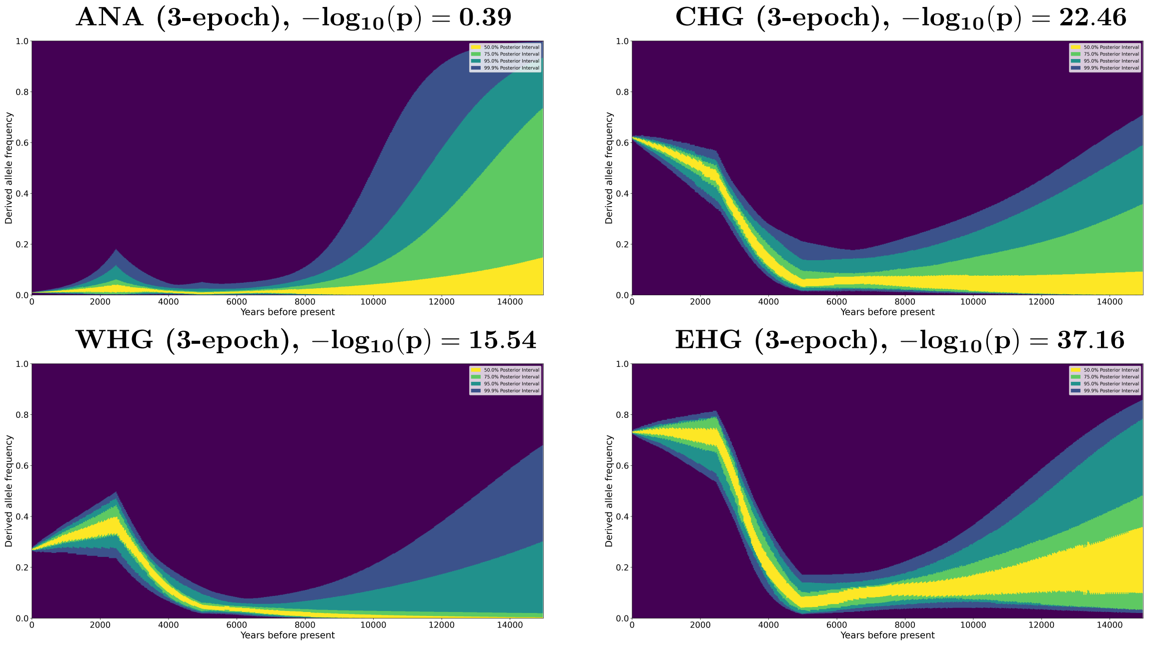

Fig S13: Ancestry-stratified allele frequency trajectories for the MCM6 SNP rs4988235, (chr2:136608646). Ancestries analyzed are Anatolian farmer (ANA), Caucasus hunter-gatherer (CHG), Western hunter-gatherer (WHG) and Eastern hunter gatherer (EHG).

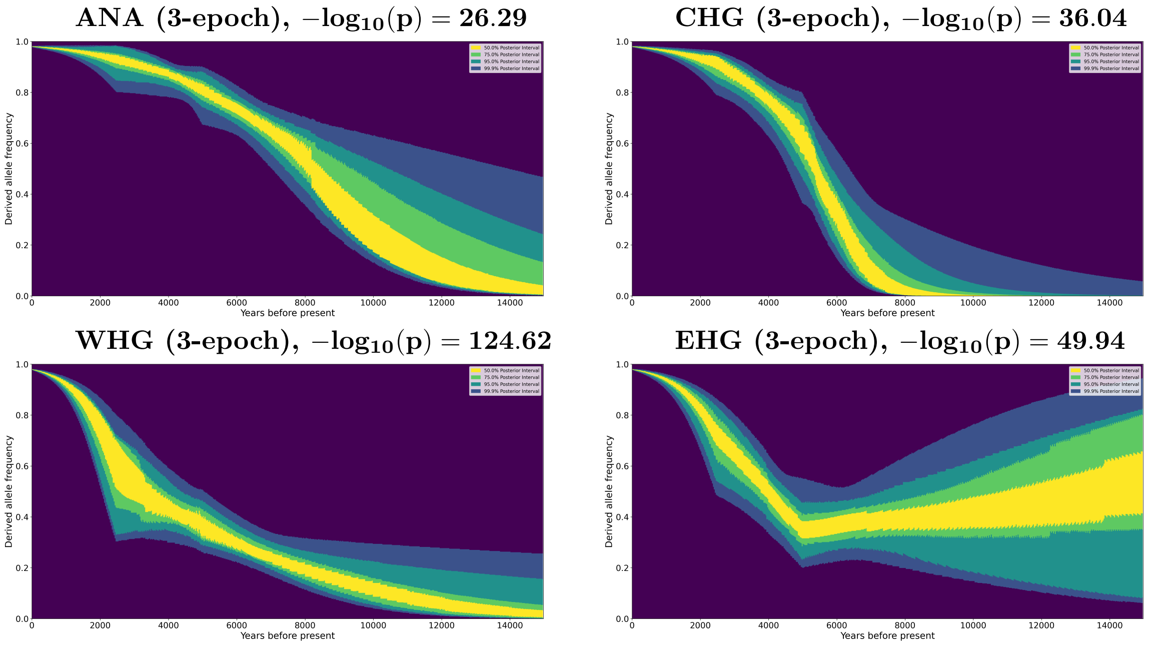

Fig S14: Ancestry-stratified allele frequency trajectories for the SLC45A2 SNP rs35395, (chr5:33948589). Ancestries analyzed are Anatolian farmer (ANA), Caucasus hunter-gatherer (CHG), Western hunter-gatherer (WHG) and Eastern hunter gatherer (EHG).

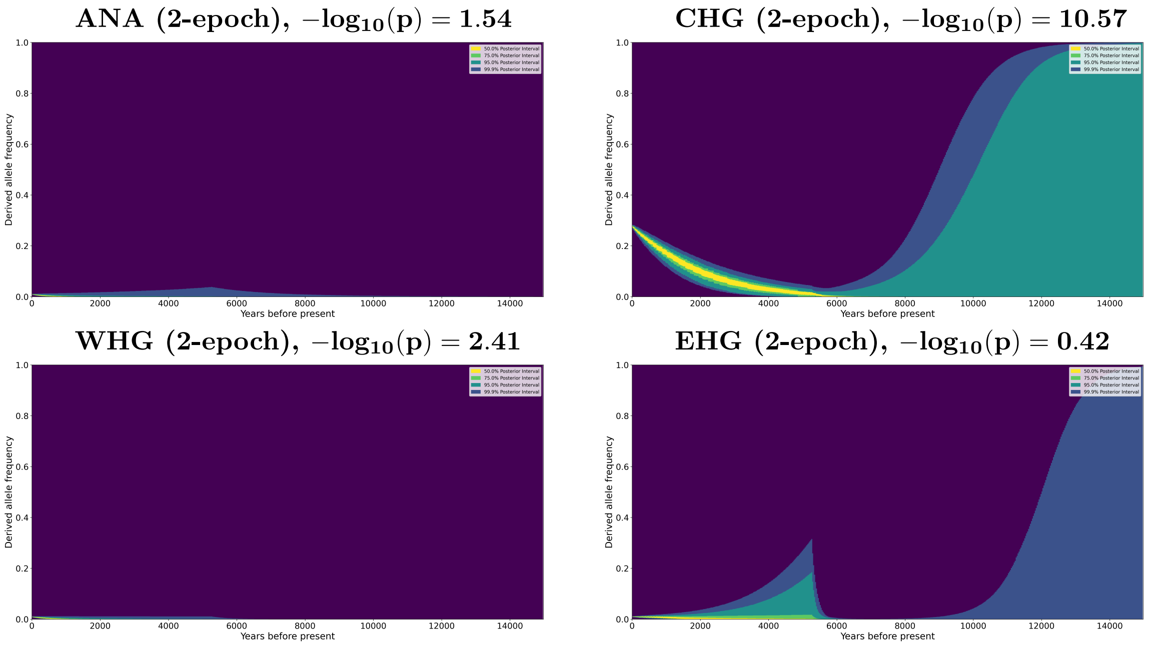

Fig S15: Ancestry-stratified allele frequency trajectories for the TNXB SNP rs12153855, (chr6:32074804). Ancestries analyzed are Anatolian farmer (ANA), Caucasus hunter-gatherer (CHG), Western hunter-gatherer (WHG) and Eastern hunter gatherer (EHG).

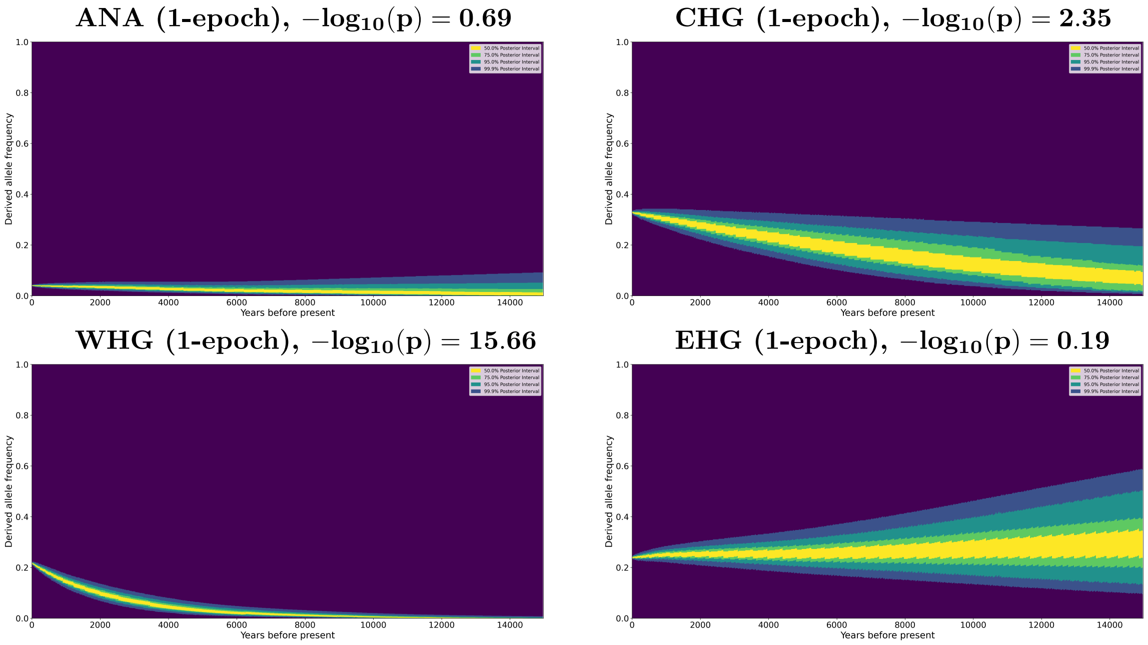

Fig S16: Ancestry-stratified allele frequency trajectories for the ACP2 SNP rs75393320, (chr11:47266471). Ancestries analyzed are Anatolian farmer (ANA), Caucasus hunter-gatherer (CHG), Western hunter-gatherer (WHG) and Eastern hunter gatherer (EHG).

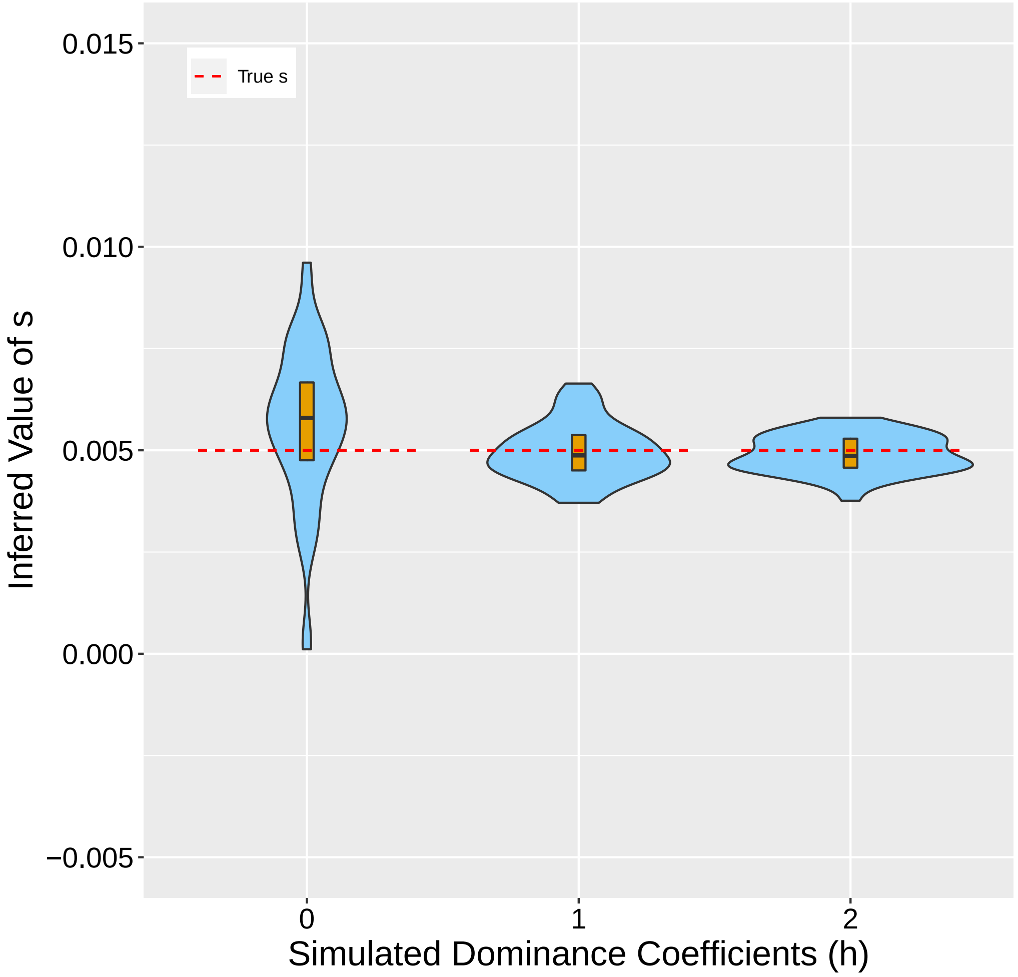

Fig S17: Violin plots showing the results of running CLUES2 on ancient genotype data with different dominance coefficients. Boxplots are overlayed with the whiskers omitted. True values of s are shown as dashed red lines. 50 replicates were performed for each dominance coefficient. A population size of $N = 50000$ was used and 8 diploid individuals were sampled in each generation.

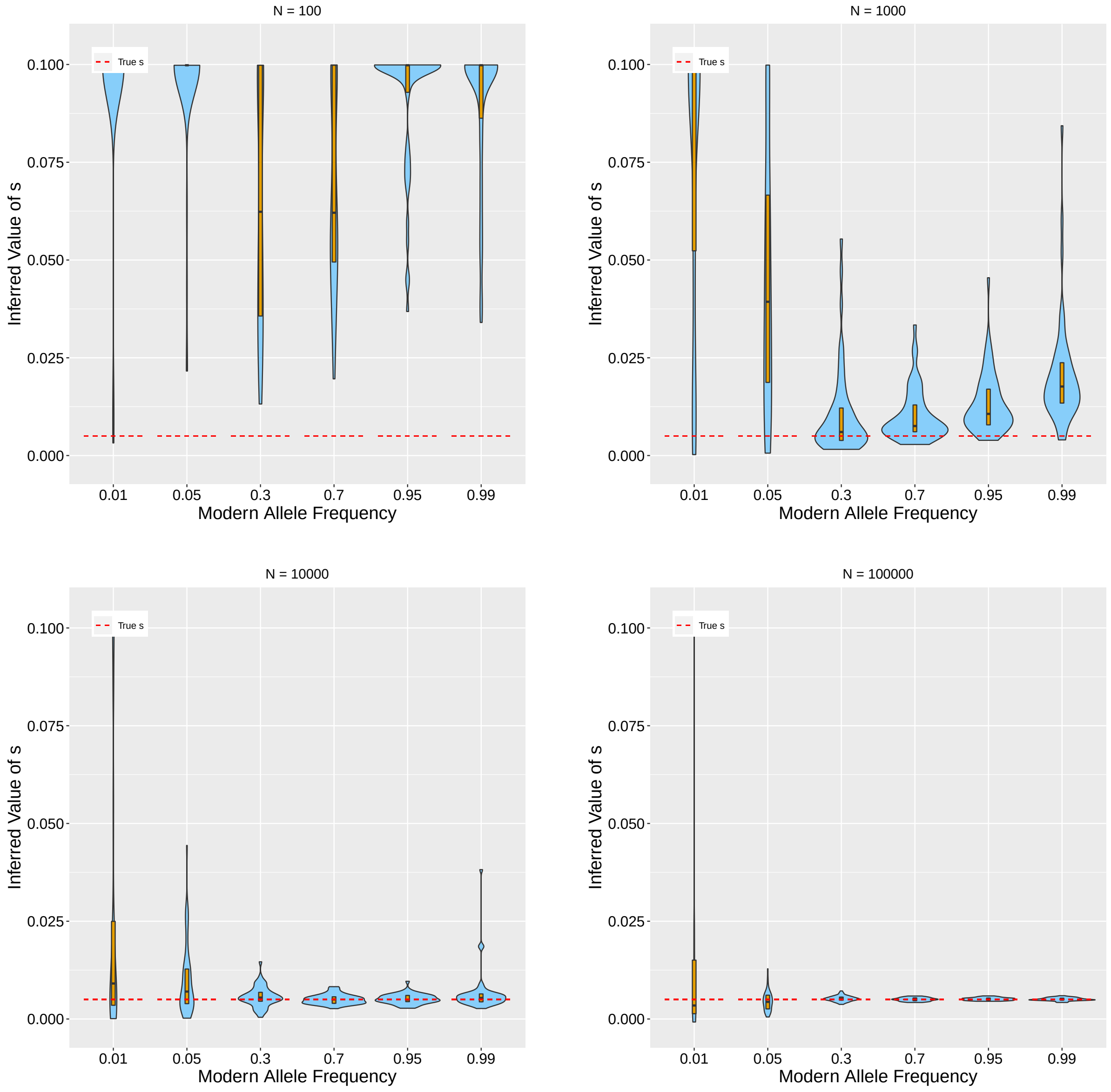

Fig S18: Violin plots showing the results of running CLUES2 on true gene trees with different modern allele frequencies and population sizes. Boxplots are overlayed with the whiskers omitted. True values of s are shown as dashed red lines. 50 replicates were performed for each combination of modern allele frequency and population size. 200 leaves were used in each simulation.

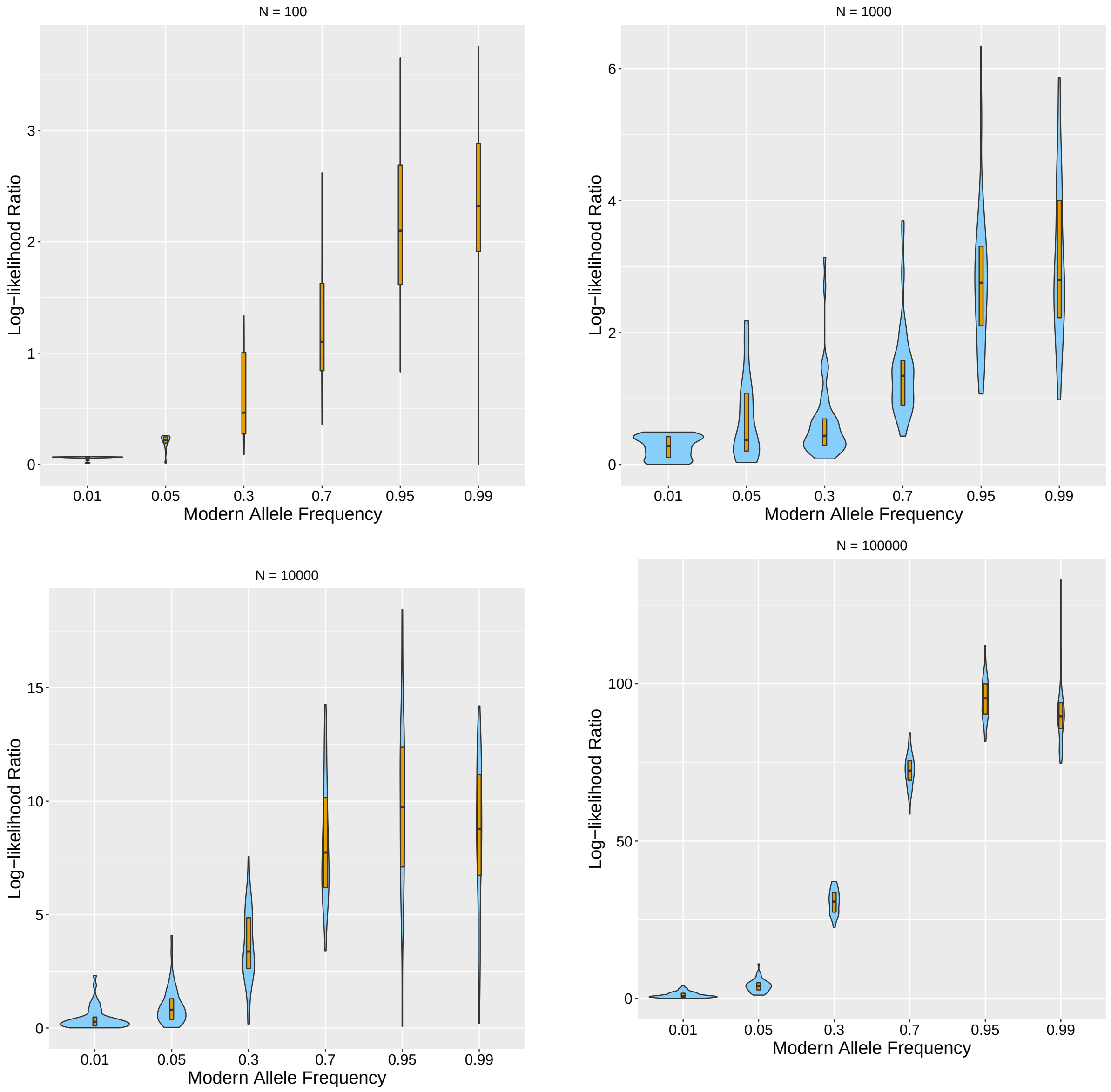

Fig S19: Violin plots showing the log-likelihood ratios against the null hypothesis $s = 0$ obtained from the simulations shown in Figure S18. Note that the ranges of the y-axis differ between the subplots.

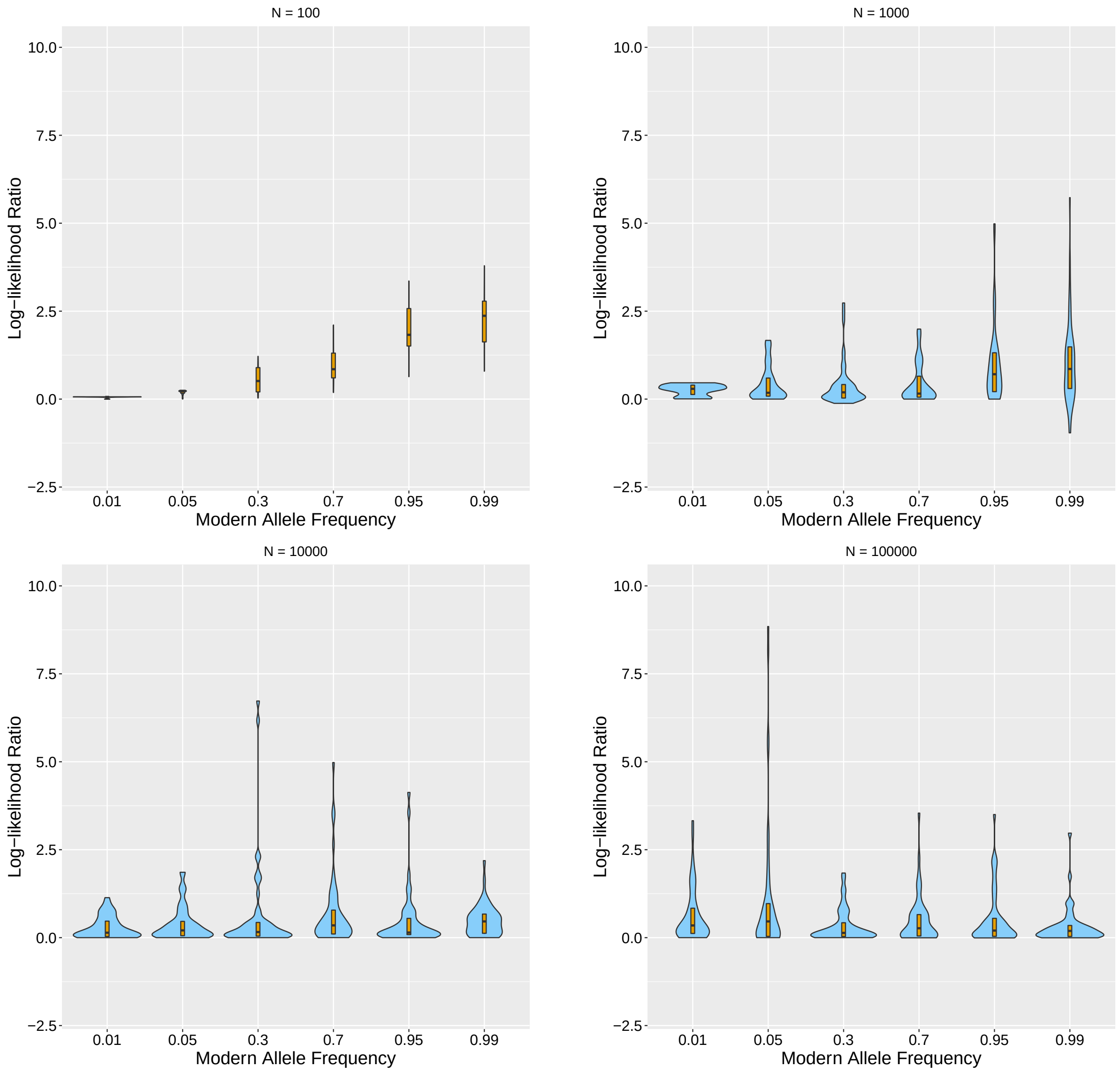

Fig S20: Violin plots showing the log-likelihood ratios against the true parameter value $s = 0.005$ obtained from the simulations shown in Figure S18.

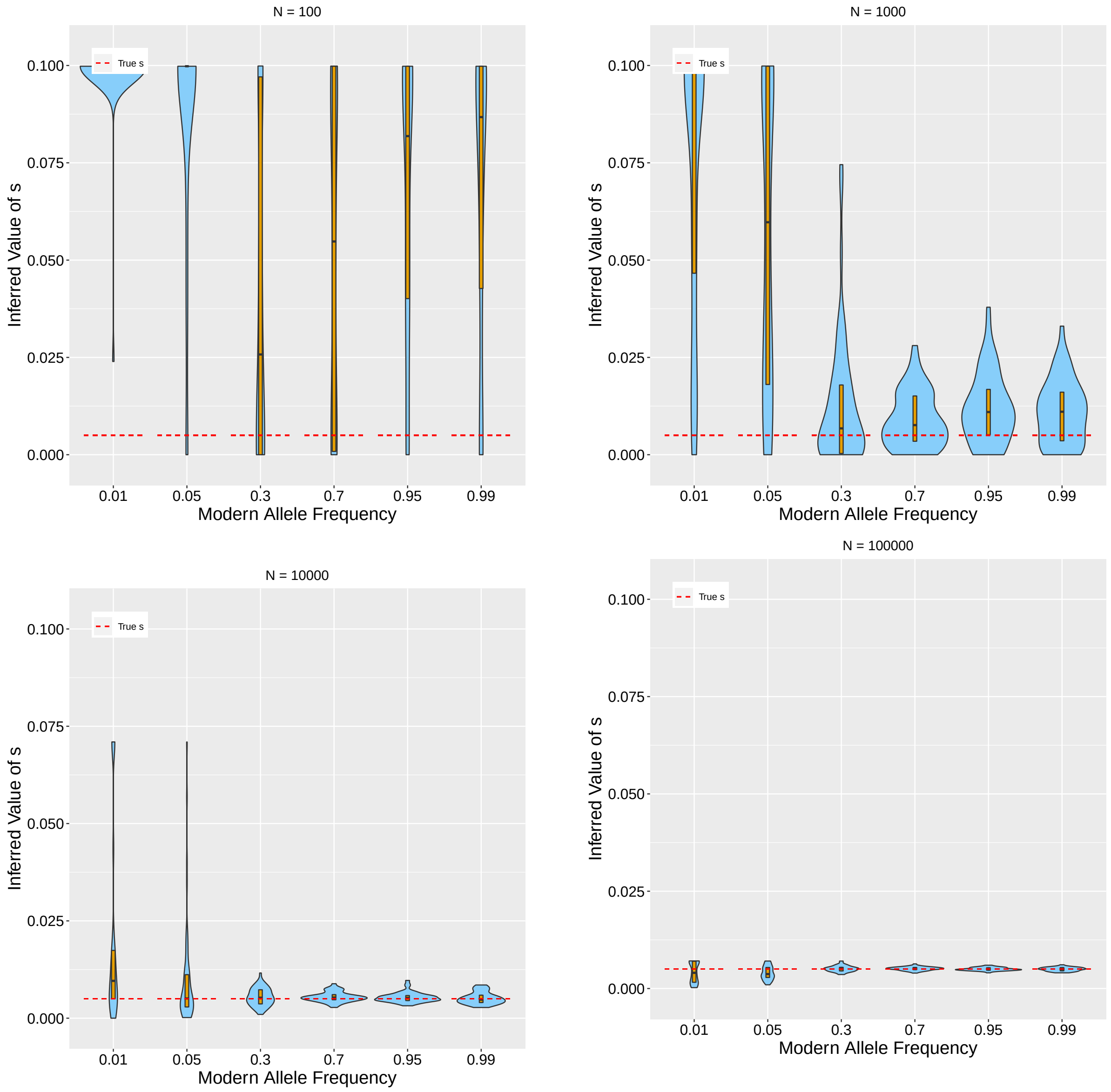

Fig S21: Results of simulations that are identical to those described in Figure S18, except that the conditional transition probability is used.

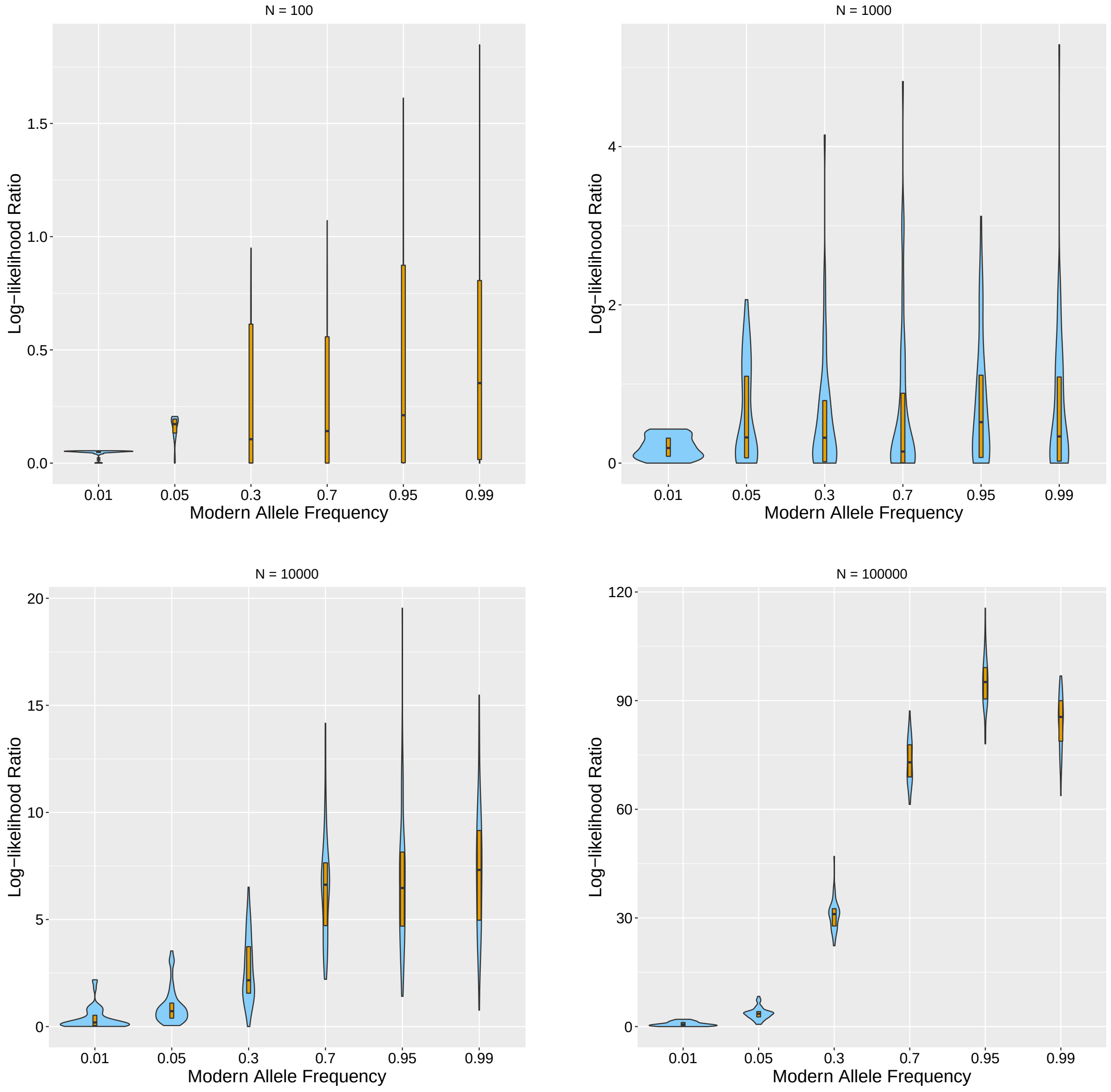

Fig S22: Results of simulations that are identical to those described in Figure S19, except that the conditional transition probability is used.

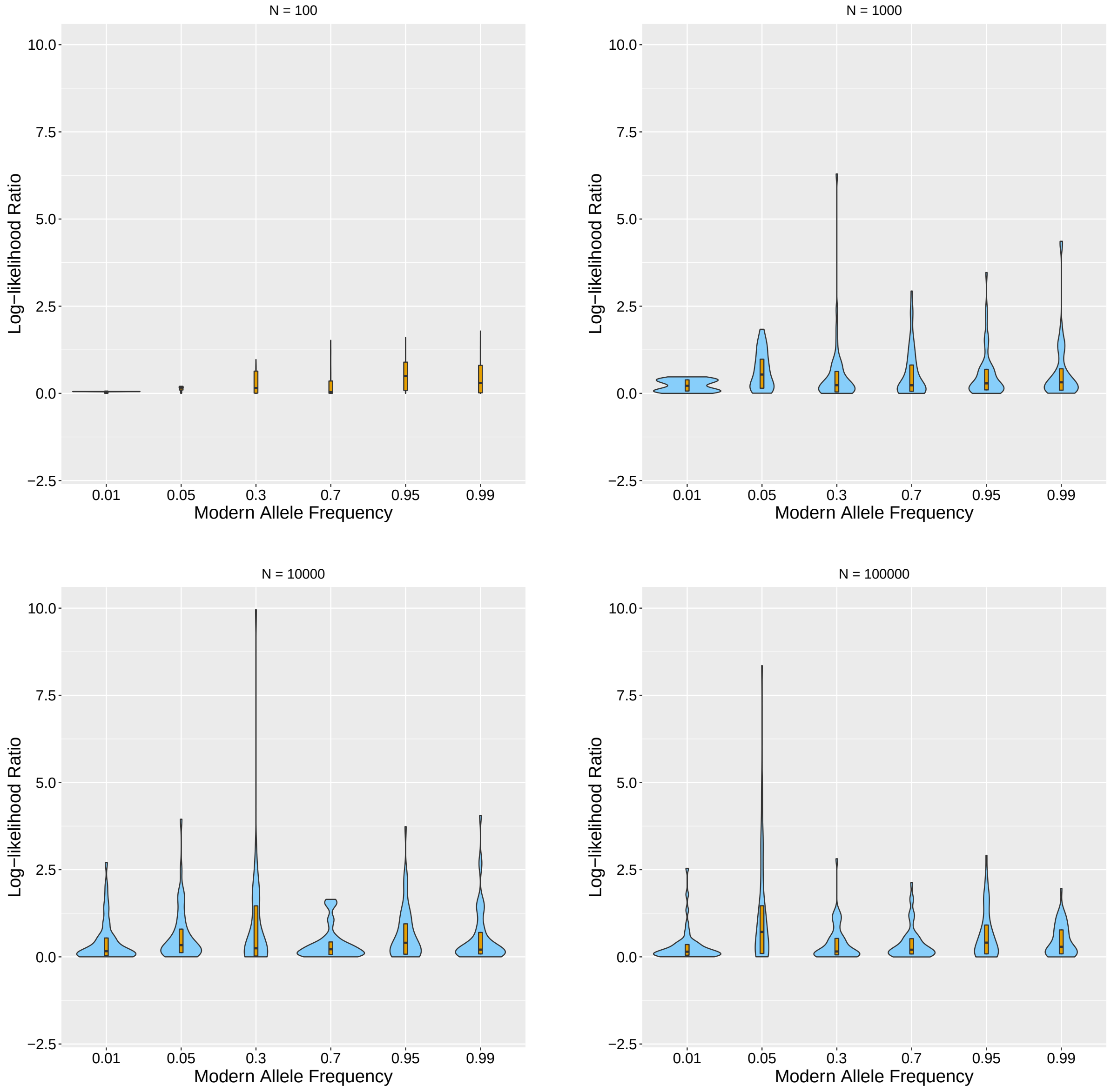

Fig S23: Results of simulations that are identical to those described in Figure S20, except that the conditional transition probability is used.
